# Identifying a parsimonious model for predicting academic achievement in undergraduate medical education: A confirmatory factor analysis

**DOI:** 10.12669/pjms.334.12610

**Published:** 2017

**Authors:** Syeda Kauser Ali, Lubna Ansari Baig, Claudio Violato, Onaiza Zahid

**Affiliations:** 1 Syeda Kauser Ali, Associate Professor, Department for Educational Development, Aga Khan University, PO Box 3500, Stadium Road, Karachi-74800, Pakistan; 2 Lubna Baig, Pro-VC and Dean, APPNA Institute of Public Health Jinnah Sindh Medical University, Rafiqui Shaheed Road Karachi, Pakistan; 3 Claudio Violato, PhD. Professor, Medical Education, Wakeforest School of Medicine, Medical Center Boulevard \ Winston-Salem, NC 27157, USA; 4 Onaiza Zahid, Research Assistant, APPNA Institute of Public Health Jinnah Sindh Medical University, Rafiqui Shaheed Road, Karachi, Pakistan

**Keywords:** MCAT, Medical Education, Psychometrics, Structural equation Modeling, Pakistan

## Abstract

**Objectives:**

This study was conducted to adduce evidence of validity for admissions tests and processes and for identifying a parsimonious model that predicts students’ academic achievement in Medical College.

**Methods:**

Psychometric study done on admission data and assessment scores for five years of medical studies at Aga Khan University Medical College, Pakistan using confirmatory factor analysis (CFA) and structured equation modeling (SEM). Sample included 276 medical students admitted in 2003, 2004 and 2005.

**Results:**

The SEM supported the existence of covariance between verbal reasoning, science and clinical knowledge for predicting achievement in medical school employing Maximum Likelihood (ML) estimations (n=112). Fit indices: χ^2^ (21) = 59.70, p =<.0001; CFI=.873; RMSEA = 0.129; SRMR = 0.093.

**Conclusions:**

This study shows that in addition to biology and chemistry which have been traditionally used as major criteria for admission to medical colleges in Pakistan; mathematics has proven to be a better predictor for higher achievements in medical college.

## INTRODUCTION

Selecting candidates most suitable for the study and practice of medicine has been a challenge for educators. It is anticipated that those selected demonstrate a readiness for medical education programs and have the potential to develop the desired professional characteristics.

The selection criteria used by different institutions comprises of assessment of cognitive and non-cognitive domains and scores on tests of prior attainment, standardized written admission tests and interviews, quality of undergraduate medical institution, referral letters, personal statements, extracurricular activities and interests, personality, motivation, language and communication skills.^1^-^5^ Effectiveness of selection has been studied by correlating the above with scholastic performance during medical school^1^-^3^ at licensing examination and during residency education.^4^,^6^,^7^

Admission tests were introduced for the first time in Pakistan in 1983 at Aga Khan University (AKU), the first private medical university in Pakistan. The AKU medical college (AKUMC) admission test (AKU-MCAT) has shown to predict scholastic performance in the first two years of medical college and demonstrate association between the system of prior education and academic performance.^8^ Applicants come from the British General Certificate of Secondary Education (GCSE) and the Pakistani Higher Secondary Certificate (HSC) system with few from other international systems.

Relationship between admission criteria and performance in medical school has been studied using correlation and regression, confirmatory factor analysis (CFA) and structural equation modeling (SEM).^9^,^10^ While correlation and regression require observed variables, CFA and SEM have the advantage of explaining the variation among both observed and latent variables.^11^

This study uses CFA and SEM for comprehensive psychometric analysis and investigation of validity evidence for three cohorts of medical students’ admission test scores and identifies a parsimonious model for predicting achievement in medical schools.

## METHODS

Data of three cohorts of students (n=276) admitted in the years 2003, 2004 and 2005 at AKUMC, graduating in 2008, 2009 and 2010 respectively was obtained from two offices; the AKU Registrar’s Office and the AKUMC Examination Cell. Records of students, on whom complete information for all years was not available, were removed from the final analysis.

The independent variables included personal data, (age, gender, place of permanent residence, system of education), total scores and subtest scores on admission test including biology, physics, chemistry, mathematics and English comprehension, evidence of prior attainment, and aggregate interviewer ratings. The dependent variables included scores on tests of knowledge of biological and clinical sciences; assessment of clinical skills and assessment of professional behaviors.

The data was analyzed using latent variable path model and assessing its fit by SEM. The relationship between observed and latent variables was construed on the basis of literature and multivariate correlations, linear regression and factor analysis using Statistical Package for Social Sciences (SPSS) version 17 for windows. Evidence of quality of admission test and medical school assessment was obtained using ITEMAN (tm) for 32-bit Windows, Version 3.6 (c) 1982 - 1998. Alternate models were derived and tested for best fit using the EQS software (a SEM program, multivariate software Inc. Copyright by PM Bentler. Version 6.1 (C) 1985 - 2010 (B97). Ethical approval was obtained from the Ethics Review Committee of the Aga Khan University.

## RESULTS

### Descriptive Analysis

A total of 276 students’ records were initially included however 49 were excluded due to missing or incomplete information at either the admission test or scores of medical college examinations, resulting in a total of 227 students included for the final analysis. Men and women were equally represented in the three cohorts with majority (97%) between the ages of 18 and 19 years.

A stable and incremental trend was seen in the number of students from British system of education (63% of those admitted in 2003 to 87% in 2005) with both GCSE Ordinary level (O-level) and Advanced level (A-level) certificates while 4-7% of those admitted studied in a mixed system. Only seven percent of the students had studied throughout in the Pakistani system of education.

The AKU-MCAT demonstrated good reliability (internal consistency) with Cronbach’s alpha ranging from 0.91 – 0.93 (Table-I). The interviewer ratings were available on a seven-point alphabetical scale. These were converted to seven-point numerical ratings where A=4, AB=3.5, B=3, BC=2.5, C=2, CD = 1,5 and D=1. Inter-rater reliability of the interview was low (r= 0.60).

**Table-I:** Descriptive analysis of the total AKUMC-AT scores.

*Class of*	*No. of examinees*	*Mean±SD*	*Cronbach Alpha*
2008	2376	73.29+17.70	0.91
2009	3171	89.25+22.03	0.93
2010	3391	90.97+22.95	0.93

Scores of the end of the year examinations in years one and two were included. Each examination included 160-170 multiple choice questions of one best type in the subjects of anatomy, physiology, biochemistry, pharmacology, pathology, microbiology and community health sciences.

Clinical knowledge was assessed by multiple choice questions targeted at assessing application of clinical knowledge in making a diagnosis, identifying investigations, proposing management plan and diagnosing complications of the disease and treatment. Assessment done at the end of six clerkships was included in the study (medicine, surgery, pediatrics, obstetrics and gynecology, psychiatry and family medicine). A descriptive analysis of the examinations administered over three years is at Table-II. All except psychiatry had moderate to good reliability (α 0.54 - 0.75) with mean item difficulty higher than 0.62.

**Table-II:** Analysis of examinations administered during the five years of medical school.

***A)Written examinations of applied basic sciences knowledge***									

	***End of year 1 examination***					***End of year 2 examination***			

	***2008***	***2009***		***2010***		***2008***	***2009***		***2010***

N of Items	159	179		174		166	160		166
Mean	113.08	135.44		108.70		113.32	109.88		116.83
Std. Dev.	10.07	15.45		11.76		14.56	10.55		8.782
Alpha	0.80	0.90		0.81		0.88	0.81		0.70

***B) Written examinations of clinical knowledge in years 3-5***											

	***Surgery***			***Medicine***			***Family Medicine***		

	***2008***	***2009***	***2010***	***2008***	***2009***	***2010***	***2008***	***2009***	***2010***

N of Items	100	100	100	100	100	100	100	100	100
Mean	66.24	64.98	70.25	63.15	78.28	71.75	72.65	75.58	74.51
Std. Dev.	6.98	7.22	5.85	7.53	5.61	6.96	6.49	4.89	4.92
Alpha	0.67	0.72	0.61	0.74	0.67	0.74	0.65	0.58	0.55

	***Obstetrics & Gyne***			***Pediatrics***			***Psychiatry***		

	***2008***	***2009***	***2010***	***2008***	***2009***	***2010***	***2008***	***2009***	***2010***

N of Items	100	100	100	100	100	100	30	30	29
Mean	67.62	74.40	71.06	69.05	75.60	80.70	24.66	24.44	22.13
Std. Dev.	5.89	5.67	5.70	6.64	4.49	5.37	2.46	1.85	1.66
Alpha	0.60	0.61	0.62	0.71	0.54	0.67	0.44	0.42	0.31

Desired professional behaviors during the clerkships were assessed using the Student Continuous Assessment Form (SCAF). The internal consistency (Cronbach’s alpha) of scores obtained by students in six major clerkships (surgery, medicine, family medicine, obstetrics and gynecology, pediatrics and psychiatry) was 0.478.

Clinical skills scores of Objective Structured Clinical Examination (OSCE) were included. The OSCE stations used a nested design and ranged from 10–16 stations depending on the objectives of the relevant clerkship.

Exploratory factor analysis was performed on 50% of the records (n=118) using composite scores of tests of clinical knowledge in the six clerkships (CK). The assessment of clinical skills and professional behaviours were also linearly added as clinical skills (CS). Principal component extraction with varimax rotation was used to decompose the correlation matrix. Five factors were identified explaining 69.30% of the variance in the data (Table-III). The rotation converged in five iterations.

**Table-III:** Principal Component Analysis with Varimax Rotation (n=118).

	*Factors*					

	*Aptitude for medicine*	*Achievement in medical school*	*Science knowledge*	*Aptitude for medicine*	*Prior attainment*	*Personal Characteristics*
Gender						0.802 (PC)
O level grades				-0.718	0.435 (PA)	
A level grades			0.634 (PA)		-0.487	
Interview ratings						-0.597
Admission committee’s rating				0.903 (AM)		
Chemistry scores	0.674(AM)					0.434
Physics scores					0.812 (AM)	
Biology scores			0.715 (SK)			
Math scores	0.984(AM)					
English scores	0.947(AM)					
Basic science examination Scores			0.767 (SK)			
Scores in assessment of clinical knowledge	0.433	0.683(AchMS)				
Scores in assessment of clinical skills		0.858(AchMS)				
Eigen values	2.69	1.98	1.69	1.58	1.35	1.24
% of Variance	19.25	14.17	12.07	11.28	9.67	8.87

AM = Aptitude for Medicine, AchMS = Achievement in Medical School, SK = Science Knowledge, PA = Prior Attainment, PC = Personal Characteristics.

A three factor model was developed for predictive validity of admission criteria for achievement in medicine. The model was based on theory of achievement and performance and used the findings on the exploratory analysis to better reflect the hypothesized directionality and relationship of the proposed latent constructs employing Maximum Likelihood (ML) estimations. The latent constructs included science knowledge, aptitude for medicine and achievement in medical school. This was fitted to the data using EQS software. In this model fit, the theoretical structure of the model was not supported with the existence of negative covariance between the latent variables of science knowledge with both achievement in medicine and aptitude for medicine. Fit indices: χ^2^ (21) = 74.57, p =<.0001; CFI=.824; RMSEA = 0.152; SRMR = 0.096.

**Fig. 1:**
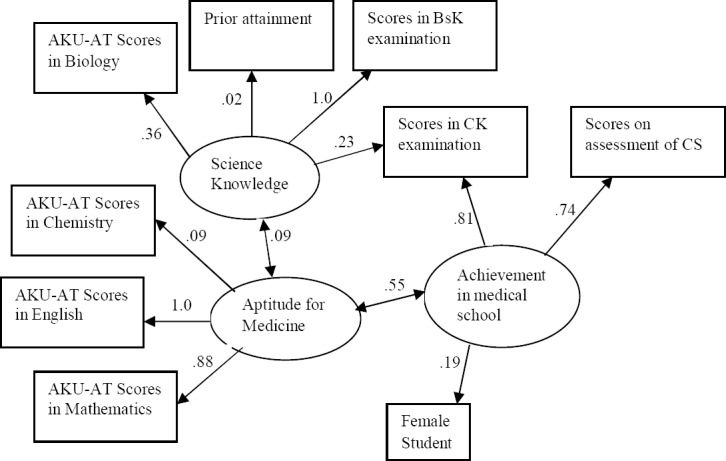
Ali and Baig’s Best Fitting Model.

An alternate model (Fig. 1) was developed where the theoretical structure and model is supported with the existence of covariance between the latent variables of science knowledge, aptitude for medicine and clinical knowledge. In this model, the combination rules of cut-off score values were achieved for the CFI at .873 and values of SRMR at .093 and RMSEA at 0.129, and a χ^2^_(21)_ = 59.70, p <.001 which was smaller than that of the earlier model. No other model came close to the fit indices reached by the alternate model and hence this was taken as the final model for factors that are effectively demonstrating predictive validity for the AKU medical college admission criteria.

## DISCUSSION

There are relatively few studies that analyse the predictive power of a variety of factors used in combination for selecting medical students.^9^,^10^ This study is an attempt to study the various cognitive and personal factors (prior achievement, admission test total and subtest scores, interview ratings and gender) for predicting performance on assessment of basic and clinical science knowledge as well as clinical skills over all five years of undergraduate medical education.

As most of the students getting admitted in AKU have early education in the GCSE system, hence the results are more comparable to studies reported by UK. McManus et al. have reported a strong correlation between A- level grades and United Kingdom Clinical Aptitude Test (UKCAT) scores as predictors of performance in undergraduate as well as postgraduate medical examinations.^4^ It is generally reported that prior academic performance accounts for a large proportion of the variance in performance in the first two years of medical school.^4^-^6^ In our study the number of A-grades in the GCSE had non-significant but positive correlation (r=.41, p >0.05) with achievement in medical school.

Predictive validity studies of the Medical College Admission Test (MCAT) of North America for the united states medical licensing examination (USMLE) report a validity coefficient of r = 0.45, *p*<.01 for Part I/Step1, r = 0.47 - 0.37 for Part II/Step 2 and r = 0.30 for Part III/Step 3.^6^,^12^ The Biomedical Aptitude Test (BMAT) introduced at the University of Cambridge, the University of Oxford, Imperial College London and University College London have shown that the scores on science sections predict first class performance in both year 1 and year 2 examination (r = 0.18 - 0.47).^13^ Studies of the Graduate Australian Medical School Admission Test (GAMSAT) reported 17% of variance in Year 1 grades.^14^,^15^ An earlier study of predictability of AKU admission test showed a significant association between admissions test scores and subject scores in the Bachelor of Surgery and Bachelor of Medicine (MBBS) Part I examinations administered at the end of year one.^8^

In this study, the sub test scores in English, Mathematics and Biology predicted overall academic achievement in medical school with validity coefficients of r=1.00, r=.88 and r=.36 respectively. While scores in Chemistry and Physics had low validity coefficients (r=0.09). Donnon et al^7^ in their meta-analysis have also reported biological sciences subtest as the best predictor of medical school performance in the preclinical years (r = 0.32, 95% CI 0.21–0.42). A positive correlation between scores on test of English language and medical school achievement is reasonable as English is the main language of instruction at AKU. The high validity coefficient of mathematics scores needs to be studied as it has not been commonly reported in literature as a predictor for medical school achievement.

The cut-off criteria used to evaluate fit indices for the final model moderately supported a three factor model of medical students’ aptitude for medical studies, science knowledge and achievement in medical school. These findings are in concordance with Violato and Donnon^16^ in which they studied 589 students’ performance on assessment of clinical reasoning skills. They got higher cut-off values for SEM model (CFI = .905, SRMR= .054, RMSEA = .105) than this study most likely because they had a larger sample size and used standardized licensing examination as their measure in addition to school level assessment, while in this study the sample size was smaller (n=227) and the measures were all at the individual school level.

### Limitations of the study

This study faced similar problems reported in earlier studies namely restriction of range, method effect and use of limited grades or ratings. In addition there was a unique problem of not having a standardized licensing examination similar to the USMLE or the Medical Council of Canada Qualifying Examinations (MCCQE) to use as a criterion. Therefore, the researchers had to use the AKU institutional examinations as the dependent variable. A larger sample size > 500 would have allowed more robust fit of the model.

## CONCLUSION

This study shows that in addition to biology and chemistry which have been traditionally used as major criteria for admission to medical colleges in Pakistan; mathematics has proven to be a better predictor for higher achievements in medical college. Mathematics is known to be associated with cognitive reasoning that would lend itself to better clinical decision making. It can be safely assumed that students with high cognitive reasoning and high score in biology perform better in medical college. Hence it is recommended that admission criteria for medical schools in Pakistan should be reviewed critically and we propose that it should include mathematics as one of the major pre-requisites.
